# High speed, complex wavefront shaping using the digital micro-mirror device

**DOI:** 10.1038/s41598-021-98430-w

**Published:** 2021-09-22

**Authors:** Ahmed B. Ayoub, Demetri Psaltis

**Affiliations:** Optics Laboratory, Ecole Polytechnique Federale de Lausanne (EPFL), 1015 Lausanne, Vaud, Switzerland

**Keywords:** Displays, Imaging and sensing

## Abstract

Digital micro-mirror devices (DMDs) have been deployed in many optical applications. As compared to spatial light modulators (SLMs), they are characterized by their much faster refresh rates (full-frame refresh rates up to 32 kHz for binary patterns) compared to 120 Hz for most liquid crystal SLMs. DMDs however can only display binary, unipolar patterns and utilize temporal modulation to represent with excellent accuracy multiple gray-levels in display applications. We used the built-in time domain dynamic range representation of the DMD to project 8-bit complex-fields. With this method, we demonstrated 8-bit complex field modulation with a frame time of 38.4 ms (around 0.15 s for the entire complex-field). We performed phase conjugation by compensating the distortions incurred due to propagation through free-space and a scattering medium. For faster modulation speed, an electro-optic modulator was used in synchronization with the DMD in an amplitude modulation mode to create grayscale patterns with frame rate ~ 833 Hz with display time of only 1.2 ms instead of 38.4 ms for time multiplexing gaining a speed up by a factor of 32.

## Introduction

SLMs have been widely used in optical applications including wave-front shaping and light focusing^1–8^. A main limitation of liquid crystal SLMs is their low refresh rates, typically less than 120 Hz. This limitation can make it impossible to achieve high performance in applications that require high speed such as beam focusing in live samples. This has opened the door for another technology, DMDs, to be used for similar applications^9–14^. As opposed to SLMs, DMDs are characterized by their much faster refresh rates. DMD devices have a maximum refresh rate of 32 kHz^15^. However, as an “on–off” device, DMD allows only binary amplitude-modulation as compared to the grayscale and phase modulation that is possible with SLMs. Despite its binary amplitude-modulation, it was shown that DMDs can outperform SLMs in beam-shaping applications^16^. In^17^, Conkey et al. demonstrated the use of Lee computer generated holograms^18^ to achieve beam focusing in scattering media with signal to background ratio up to ~ 160 in ~ 34 ms. An improved optical performance was achieved by projecting Lee holograms on the DMD along with spatial filtering and misaligned optical lenses for phase modulation using “Super-pixel” technique where each super-pixel is composed of multiple pixels of the DMD^19^. In^20^, Drémeau et.al used the DMD as a binary input in a reference-less optical system for beam focusing. A Bayesian phase retrieval algorithm^21^ was used to precisely estimate the transmission matrix as well as for beam focusing through a white paint layer which acted as the highly scattering medium. In recent years^22^, Wang et.al implemented a binary-based digital optical phase conjugation (DOPC) system based on the use of DMD instead of SLMs. They used phase retrieval methods to design a binary computer generated hologram and demonstrated speed enhancement by approximately 2 orders of magnitude as compared to a liquid crystal SLM with a low playback latency of ~ 5 ms.

In display application, the DMD is used to modulate the time integrated light intensity through sequential pulse width modulation^23–26^. In the context of optical diffraction tomography^27^, Lee et.al demonstrated a time-multiplexing structured illumination control scheme using DMDs^28^. In their paper, the authors showed an improved performance by using time-multiplexed 8-bit amplitude modulation as compared to binary amplitude modulation (i.e. Lee holograms) in which the unwanted artifacts were diminished.

In this paper, we use a DMD to perform complex (amplitude and phase) modulation. We used the built-in dynamic range feature of the DMD device and to control the frame time of the DMD, to project an 8-bit image in 38.4 ms (256 × 150 µs). We show that by splitting the complex field into real and imaginary parts and using the time modulation scheme of the DMD we were able to synthesize a complex signal. We demonstrated this method for wavefront shaping by phase conjugating a measured field in free space and/or passing through a scattering medium. This paves the way for imaging and optical phase conjugation systems that are fast, digital accuracy without compromising light efficiency. Finally, to increase further the frame rate for grayscale modulation using the DMD, we modulated the intensity of the illuminating beam for each bit-plane while fixing the time for each bit-plane. Using this modulation scheme, the frame time for each grayscale image was reduced to 1.2 ms (8 × 150 µs).

In what follows, we first introduce the basic concept we use in the paper. After that, in the next sub-section in the methods section, we show how we used this technique for phase conjugation applications. We refer to this method as Opto-Electronic Phase Conjugation (OEPC) to distinguish it from conventional Digital Phase Conjugation. The results section was divided into 2 main sub-sections. The first subsection includes two experiments that demonstrate phase conjugation conducted using our time-multiplexed technique. The second sub-section shows a new modality for using the DMD which is amplitude modulation scheme.

## Methods

### Complex wavefront shaping with the DMD

We consider a complex field $f(x,y) = A(x,y)e^{j\varphi (x,y)}$ where $A(x,y)$ is the 2D amplitude and $\varphi (x,y)$ is the 2D phase pattern. We represent a complex pattern on the DMD by presenting positive and negative portions of real and imaginary parts of the complex field, sequentially. In order to represent the grayscale of the real and imaginary parts of the complex field, we take advantage of the time modulation feature of the DMD. $f(x,y)$ can be written as follows:

1 $$ \begin{aligned} f(x,y) & = {\text{Re}} \{ f(x,y)\} + i*{\text{Im}} \{ f(x,y)\} \\ & = \sum\limits_{\alpha = 1}^{8} {BR_{\alpha } 2^{\alpha - 1} + } i*\sum\limits_{\alpha = 1}^{8} {BI_{\alpha } 2^{\alpha - 1} } \\ \end{aligned} $$

where $BR_{\alpha }$ and $BI_{\alpha }$ are the $\alpha$ bit-planes for the real and imaginary parts, respectively. In our optical system, the field is interferometrically measured at the detector. This allows us to reconstitute the complete complex field by temporal integration on the detector which aggregates the contributions of each of the bit planes of both real and imaginary parts. Alternatively, the summation can be done post-detection on the computer.

**Figure 1:**
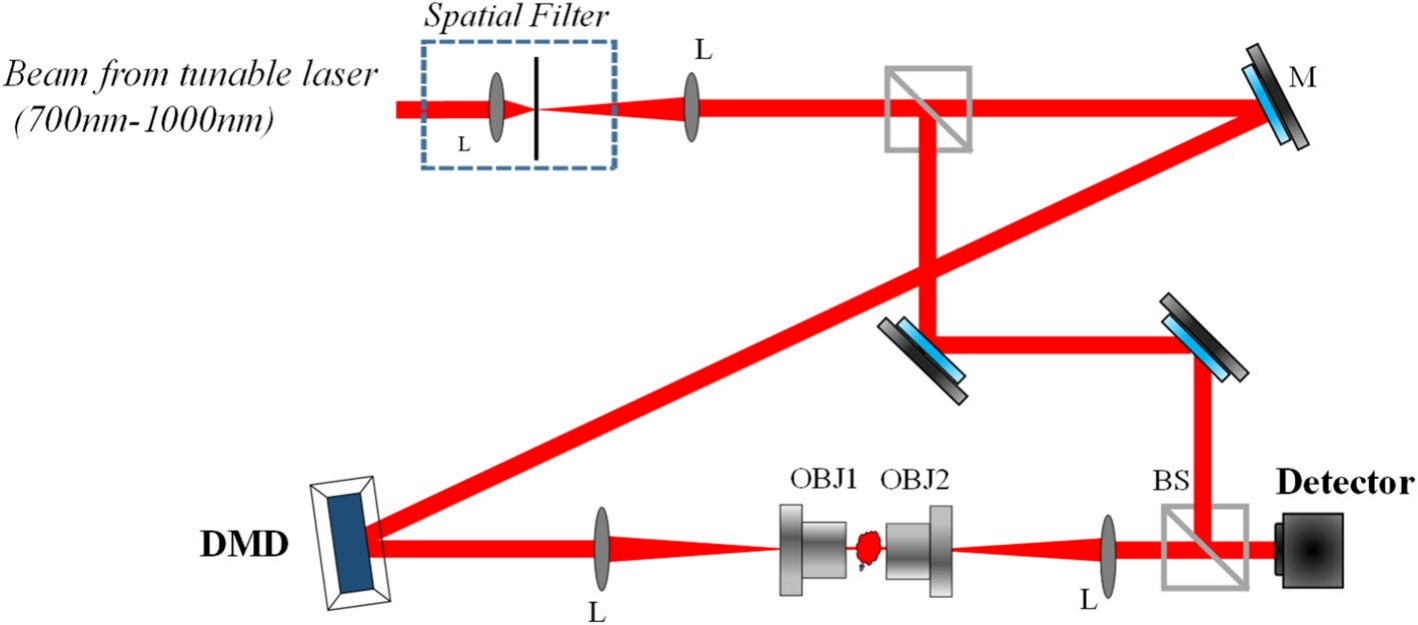
Optical setup. (*M* mirror, *L* lens, *OBJ* objective lens, *BS* beam splitter). 4f systems are used to image DMD pattern onto the detector with a spatial magnification defined by the 4f systems.

We used the experimental setup shown in Fig. 1.

A continuous wave (CW) laser at 725 nm was used in all our experiments. The laser beam is spatially filtered and collimated with a pinhole and a pair of lenses. The signal beam is reflected from the DMD device (DLP4500NIR 0.45 WXGA near-Infrared, resolution: 912 × 1140, pixel pitch: 10.8 µm, Ajile Light Industries). A 4f. system was used to image the DMD plane onto the detector plane to retrieve in-focus images. For holographic detection, the signal and reference beams are combined using a non-polarizing beam splitter and projected onto a complementary metal–oxide–semiconductor (CMOS) camera (Edmund optics, pixel size = 3.45 µm, resolution = 2056 × 1542 pixels). Although in the current experiment there is no object to be imaged, objective lenses were used to allow the DMD to be used for imaging applications.

**Figure 2:**
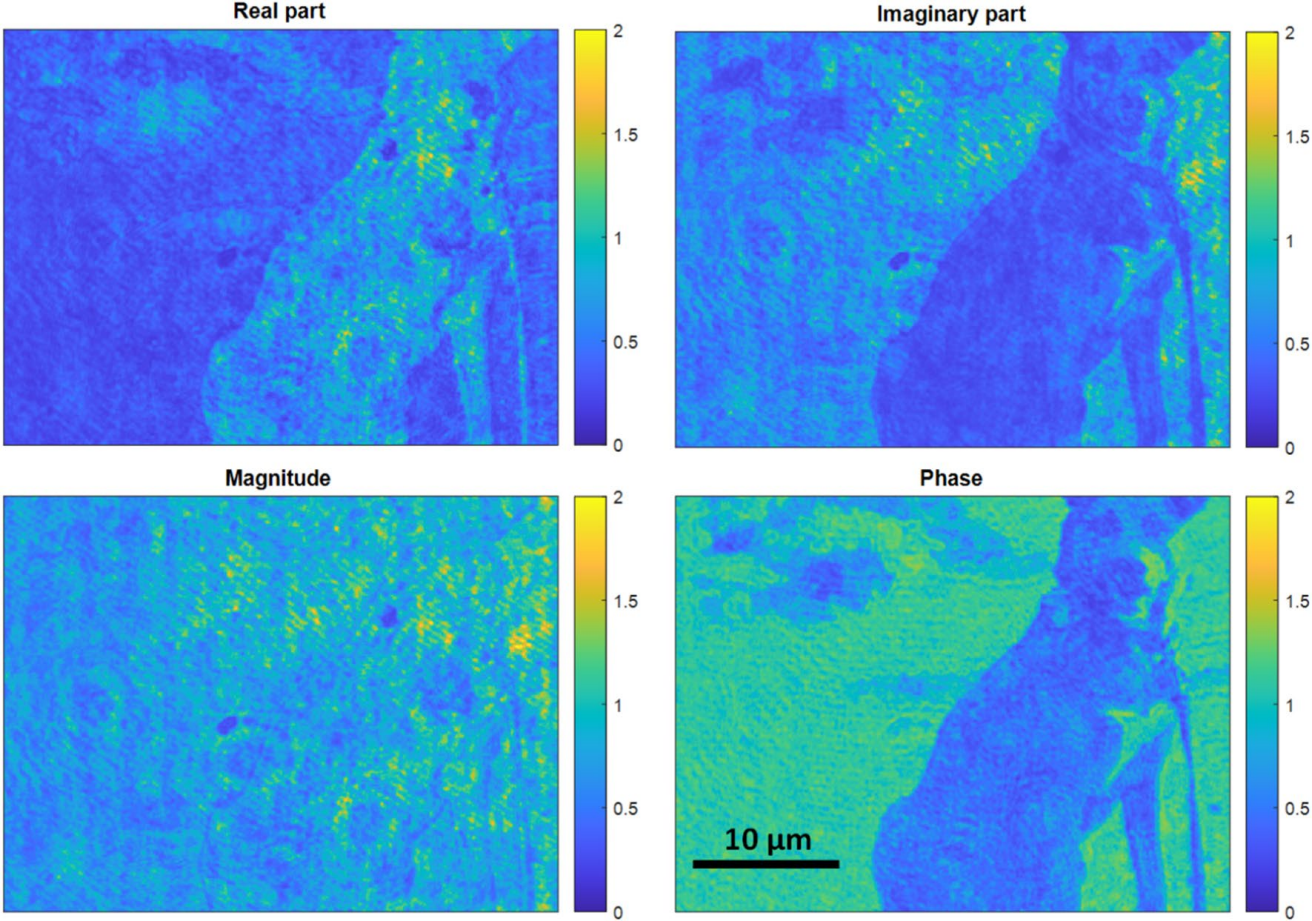
Phase modulation using DMD as expressed in equation (1) by splitting the complex field into real and imaginary parts.

The grayscale image was translated into 8 binary bit-planes (corresponding to the 8-bit image) and the 8 binary images were displayed onto the DMD sequentially to be captured onto the detector within a limited exposure time in a time-multiplexing manner. Since time-multiplexing is used, this means that the least significant bit-plane will be displayed for 1 time unit while the most significant bit-plane will be displayed for 128 time units (i.e. 1 time unit = 150 µs). This was repeated for both the real and the imaginary parts of the image. Figure 2 demonstrates successful phase modulation as a result of this technique. Each of the real and the imaginary parts were projected, one at a time, onto the DMD in a time multiplexing fashion. After capturing the two grayscale mages of the real and the imaginary parts, they were added digitally onto the computer which is equivalent to phase modulation by the DMD as seen in Fig. 2.

For phase maps whose phase exceeds π/2 radians, we split the real and imaginary parts into positive and negative values and displayed each one separately onto the DMD. This is expressed by Eq. (2):

2 $$ \begin{aligned} f(x,y) & = {\text{Re}} \{ f(x,y)\} + i*{\text{Im}} \{ f(x,y)\} \\ & = \left[ {\sum\limits_{\alpha = 1}^{8} {BR_{\alpha }^{p} 2^{\alpha - 1} } - \sum\limits_{\alpha = 1}^{8} {BR_{\alpha }^{n} 2^{\alpha - 1} } } \right] \\ & \quad + i*\left[ {\sum\limits_{\alpha = 1}^{8} {BI_{\alpha }^{p} 2^{\alpha - 1} } - \sum\limits_{\alpha = 1}^{8} {BI_{\alpha }^{n} 2^{\alpha - 1} } } \right] \\ \end{aligned} $$

where $BR_{\alpha }^{p}$ and $BR_{\alpha }^{n}$ are the $\alpha$ bit-planes for the positive and negative parts of the real component, respectively, while $BI_{\alpha }^{p}$ and $BI_{\alpha }^{n}$ are the $\alpha$ bit-planes for the positive and negative parts of the imaginary component, respectively.

### Opto-electronic phase conjugation (OEPC) using DMD

Assuming an initial complex field ($U_{i}$) incident on a scattering medium, we can write the output as follows:

3 $$ U_{o} = T_{io} U_{i} $$

where $T_{io}$ is the transmission matrix describing the propagation of $U_{i}$ through the scattering medium. After taking the complex conjugate of this function and propagate through the same medium, we get:

4 $$ \begin{aligned} U_{final} & = T_{oi} U_{o}^{*} \\ & = T_{oi} (T_{io} U_{i} {)}^{*} = T_{oi} T_{io}^{*} U_{i}^{*} \\ \end{aligned} $$

Assuming a perfect time-symmetric system (i.e. $T_{oi}^{ - 1} = T_{io}^{*}$), Eq. 4 simplifies to:

5 $$ U_{final} = T_{oi} T_{io}^{*} U_{i}^{*} { = }T_{oi} T_{oi}^{ - 1} U_{i}^{*} = \, U_{i}^{*} $$

**Figure 3:**
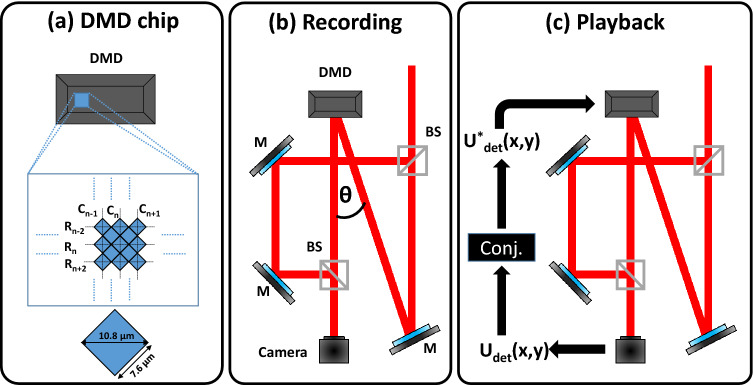
OEPC optical setup. (**a**) DMD projector grid showing the pixel orientation. (**b**) Recording step is performed first to record the hologram after free-space propagation. The second step is the (**c**) playback where the complex conjugate of the detected field is displayed onto the DMD and the original field is retrieved. *M* mirror, *BS* beamsplitter.

Equation (5) shows how OEPC removes the aberrations associated with the medium (i.e. free space propagation or mouse liver tissue in our experiments) and the input field is retrieved. In order to demonstrate OEPC using the DMD, a simplified interferometric setup was used as shown in Fig. 3. The detector used in this experiment is a CMOS camera (Photonfocus Inc., resolution: 1312 × 1280, pixel size: 8 µm). First, the setup was calibrated to ensure pixel-to-pixel alignment between the DMD and the camera. The incident angle is chosen to be at θ degrees with respect to the direction normal to the DMD surface. Given that the DMD has an “on” and “off” state beam at ± 12° with respect to the normal direction, θ was chosen to be around 12° relative to the normal direction as shown in Fig. 3b. Owing to the specific distribution of the pixels in the DMD, (Fig. 3a), interpolation was used in order to successfully align the pixels of the DMD and SLM. As seen in Fig. 3a, the center-to-center distance between two successive columns (i.e. C_n−1_ and C_n_) is 10.8 µm while the distance between two successive rows is equal to 5.4 µm (i.e. 10.8/2 µm) as given by the DMD specification. This difference was accommodated by the use of interpolation to ensure successful mapping of the detector image onto the DMD screen. After calibrating the system, the recording step (Fig. 3b) was performed to by displaying phase and/or amplitude maps as expressed by Eq. (2). After acquiring the image on the CMOS detector, the hologram was processed and the complex field was extracted on a personal computer and its complex conjugate was displayed back onto the DMD (Fig. 3c) and finally the original non-distorted image is reconstructed by adding the real and the imaginary parts digitally.

## Results

### OEPC for diffracted pattern after passing through scattering media

**Figure 4:**
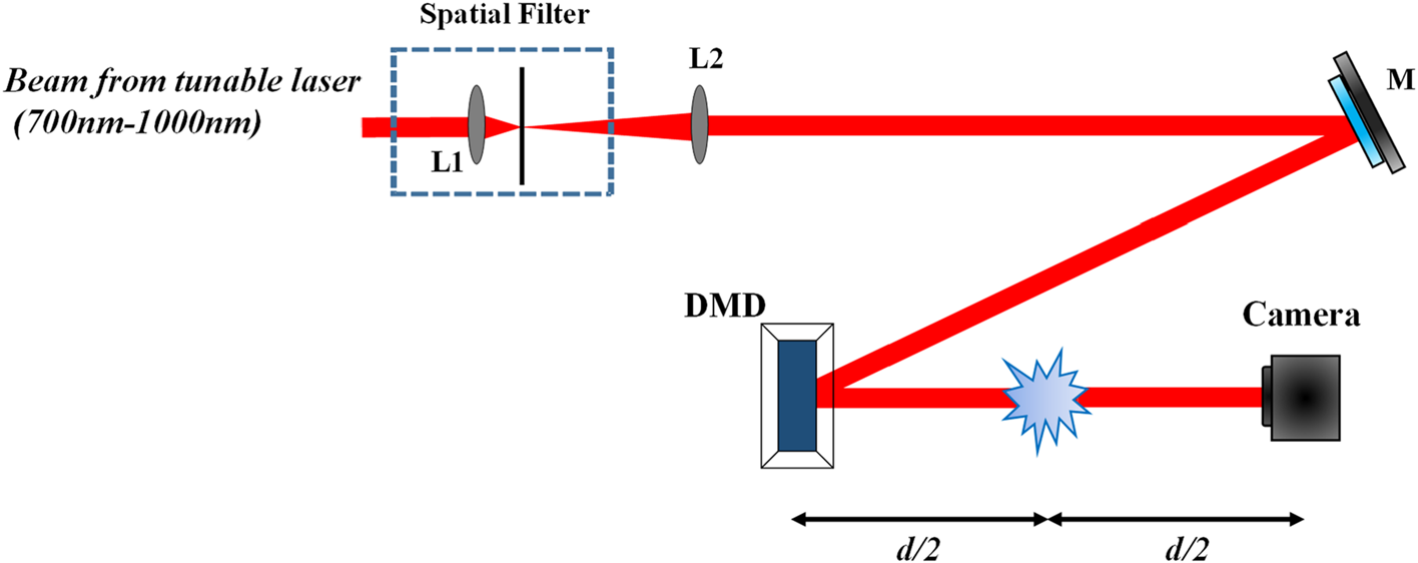
Experimental setup with the scattering medium inserted in between the DMD and the camera (*M* mirror, *L1&L2* lenses, d = 28 cm).

An experiment was performed with a scattering medium in between the DMD and the detector as shown in Fig. 4. The scattering medium was a 10 µm-thick fixed mouse liver-tissue sandwiched between a standard cover-slide of 1 mm thickness and a coverslip of 170 µm thickness.

**Figure 5:**
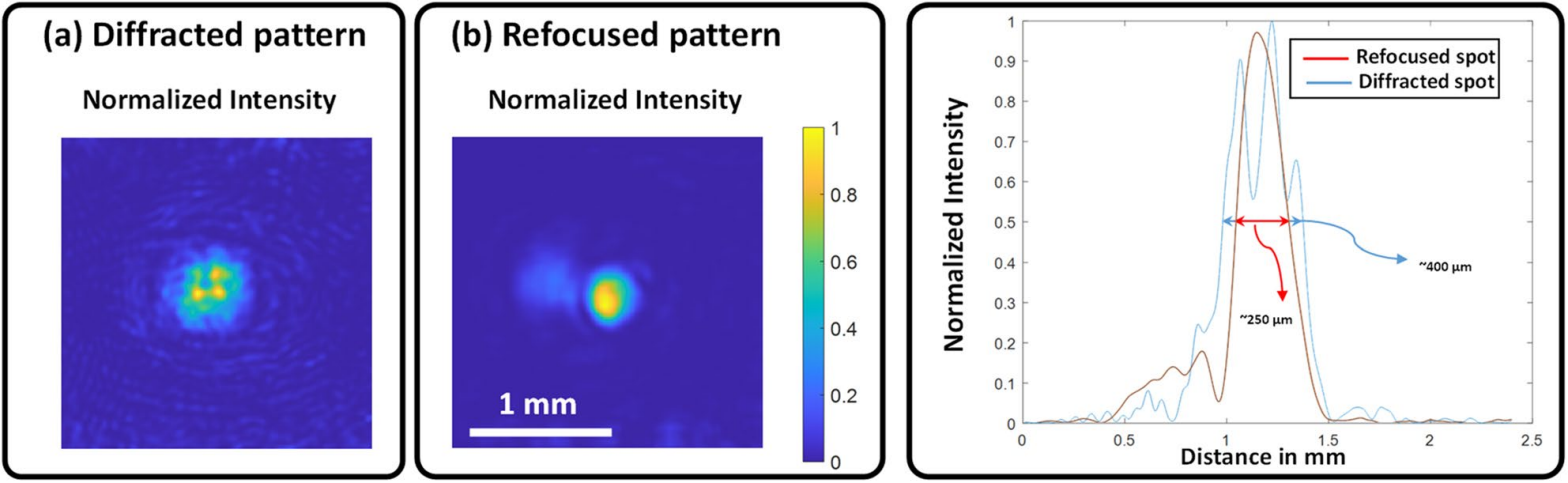
Intensity pattern before and after refocusing using OEPC technique. (**a**) Diffracted intensity pattern after propagating through the scattering medium path while (**b**) shows the intensity pattern after OEPC.

In the OEPC experiment, a simple object (a binary amplitude circular mask of ~ 430 µm diameter) was displayed on the DMD and the reflected light was captured by the detector. By extracting the complex field of the acquired hologram, taking its complex conjugate and feeding it back to the DMD with the scattering medium in place, we were able to retrieve the original pattern. Figure 5 shows the diffracted and the refocused spot after OEPC. Figure 5b shows how we get a full-width at half-maximum (FWHM) of 250 µm whereas the defocused spot (Fig. 5a) has a FWHM of 400 µm.

**Figure 6:**
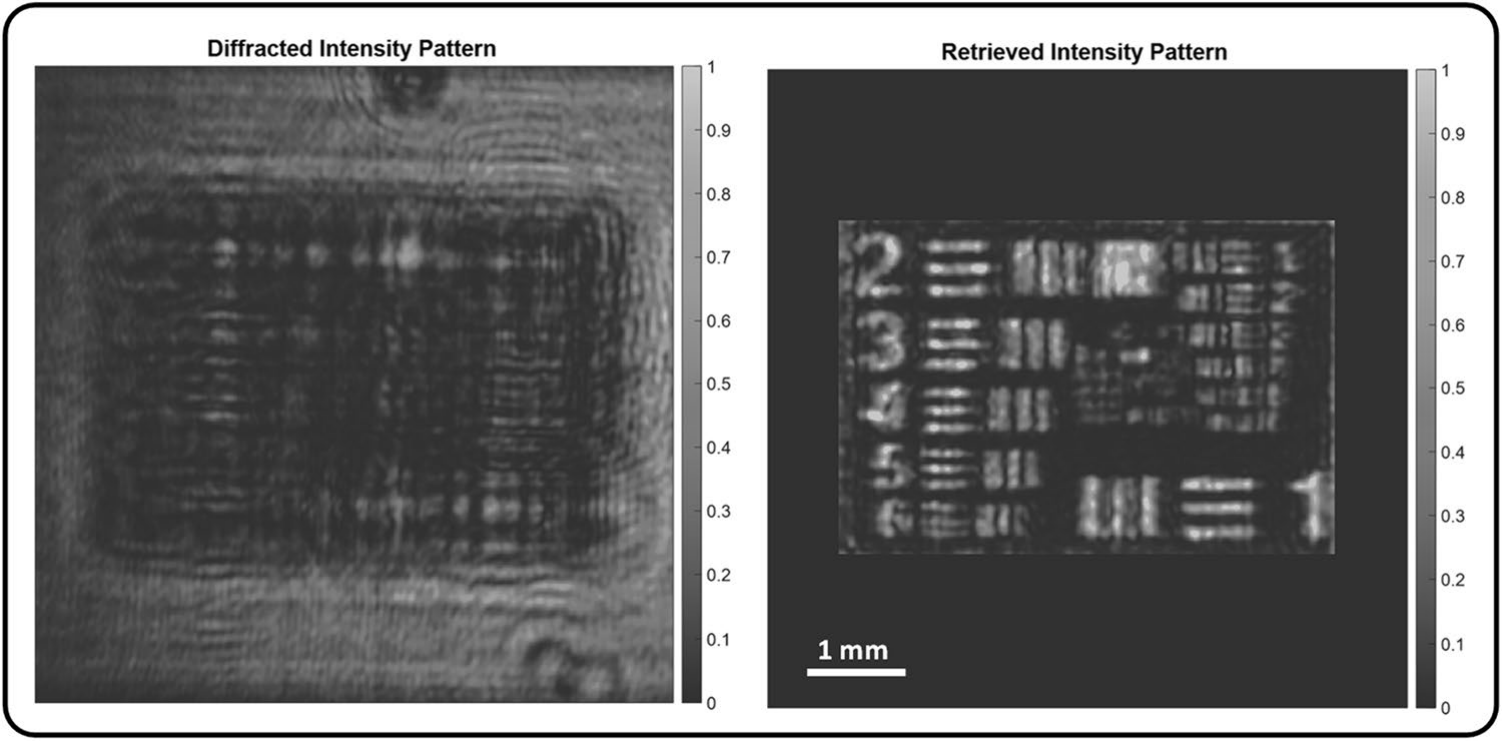
Intensity pattern before and after refocusing using OEPC. Left: diffracted intensity pattern after propagating through the scattering medium. Right: shows the intensity pattern after OEPC.

A similar experiment was performed with the USAF target being the input amplitude-only field passing through the same scattering medium (i.e. liver tissue). The image on the left hand-side of Fig. 6 shows the diffracted intensity pattern through the sample while the right hand-side image shows the refocused USAF target after compensating the effect of defocusing and scattering through the sample.

### Grayscale modulation using amplitude modulator synchronized with the DMD

**Figure 7:**
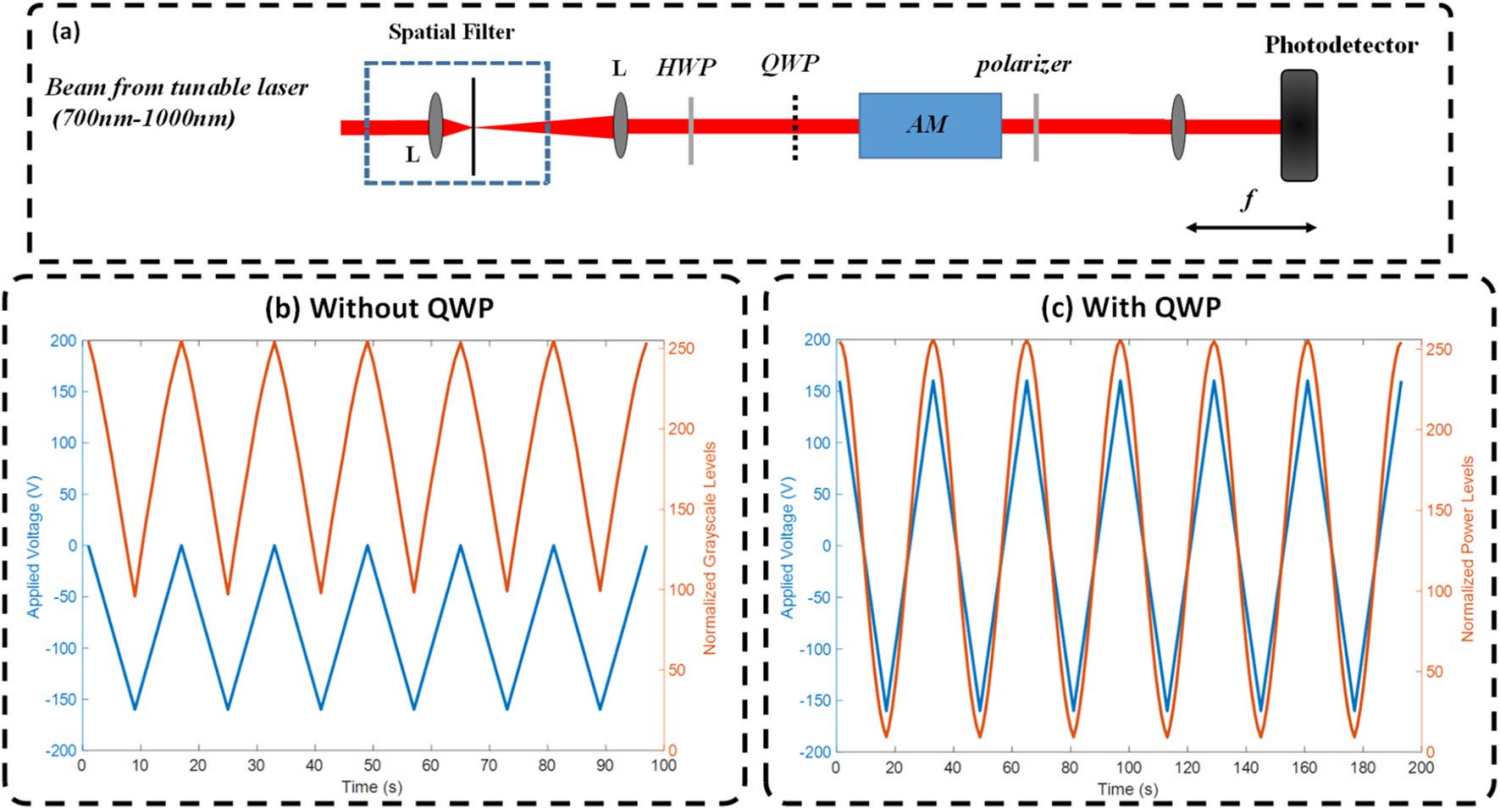
Amplitude modulation. (**a**) Calibration system for the amplitude modulator. (**b**) Amplitude modulation (orange) as a function of the applied voltage (blue) without the QWP. (**c**) Amplitude modulation (orange) as a function of the applied voltage (blue) with the QWP. Comparing (**b**) and (**c**), adding the QWP resulted in creating an input circular polarization which resulted in getting the complete modulation range.

In order to boost further the speed of grayscale modulation using the DMD, amplitude modulation was used instead of time modulation. As shown in Fig. 7, an electro-optic modulator (Thorlabs, EO-AM-NR-C1) was used to modulate the light intensity for each bit-plane. In this case we used light intensity instead of light amplitude since holographic recording was not used on the camera. Therefore the calibration was done for intensity recording. The NI PCIe-6321 data acquisition tool was used for fast synchronization permitting speeds up to 90 kHz which is much faster than the DMD speed (6.6 kHz). Figure 7 shows the calibration process of the amplitude modulator (AM). A photodetector (Thorlabs, PDA36A-EC) was used to measure the voltage signal from the AM as a function of the applied voltage. As the characteristic half-wave voltage (V_π_) at 725 nm for the AM was around 240 V, high voltage was needed to get the full modulation range which was generated using a high voltage amplifier (HVA200, V_in_ = − 10:10, V_out_ = − 200:200, Thorlabs). A quarter wave plate (QWP) was used to get the full range since V_π_ is higher than the maximum possible voltage generated by the voltage amplifier.

**Figure 8:**
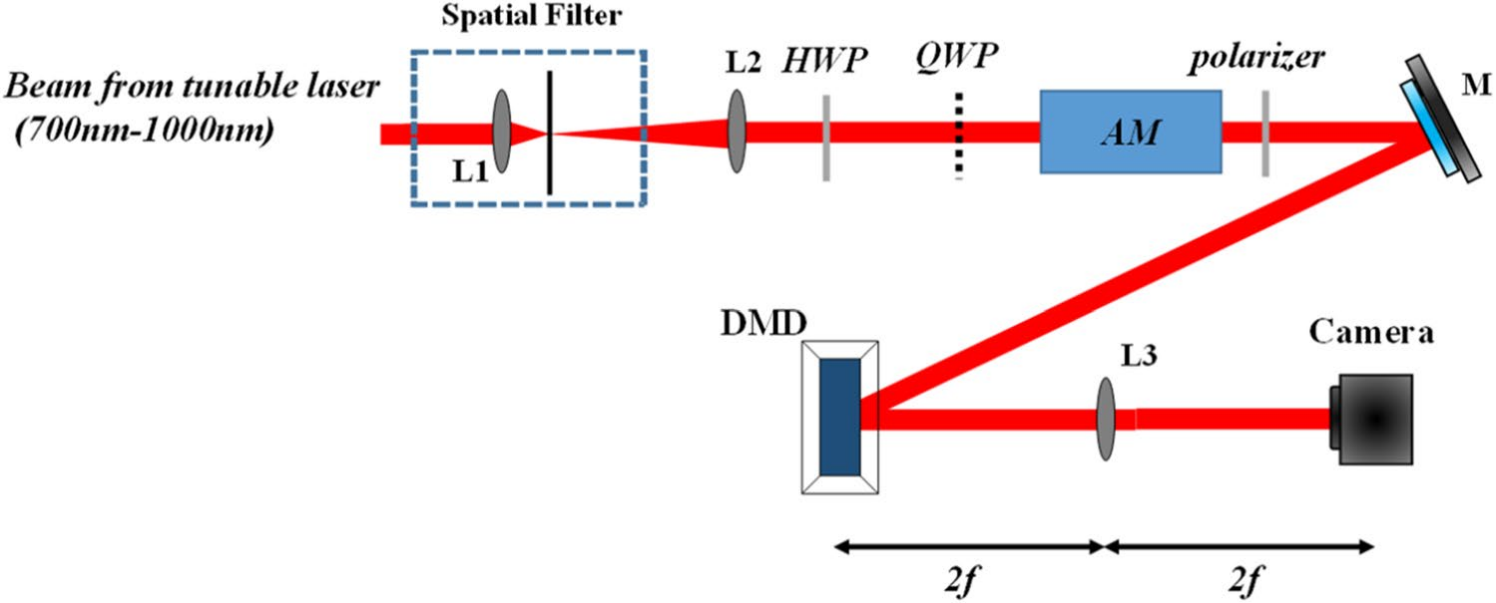
Amplitude modulation using AM synchronized with the DMD.

A look-up-table (LUT) is extracted from Fig. 7c to modulate the 8 bit-planes of the 8-bit grayscale image. After calibrating the AM, the amplitude modulator was synchronized with the DMD and images were recorded using the camera as shown in Fig. 8. With such modulation scheme a maximum achievable frame rate of 833 Hz could be reached (i.e.$\frac{1}{{150\;\upmu {\text{s}} \times 8}}$). Using this amplitude-modulation scheme, we showed efficient grayscale modulation for sinusoidal grating using the DMD (see supplementary video).

**Figure 9:**
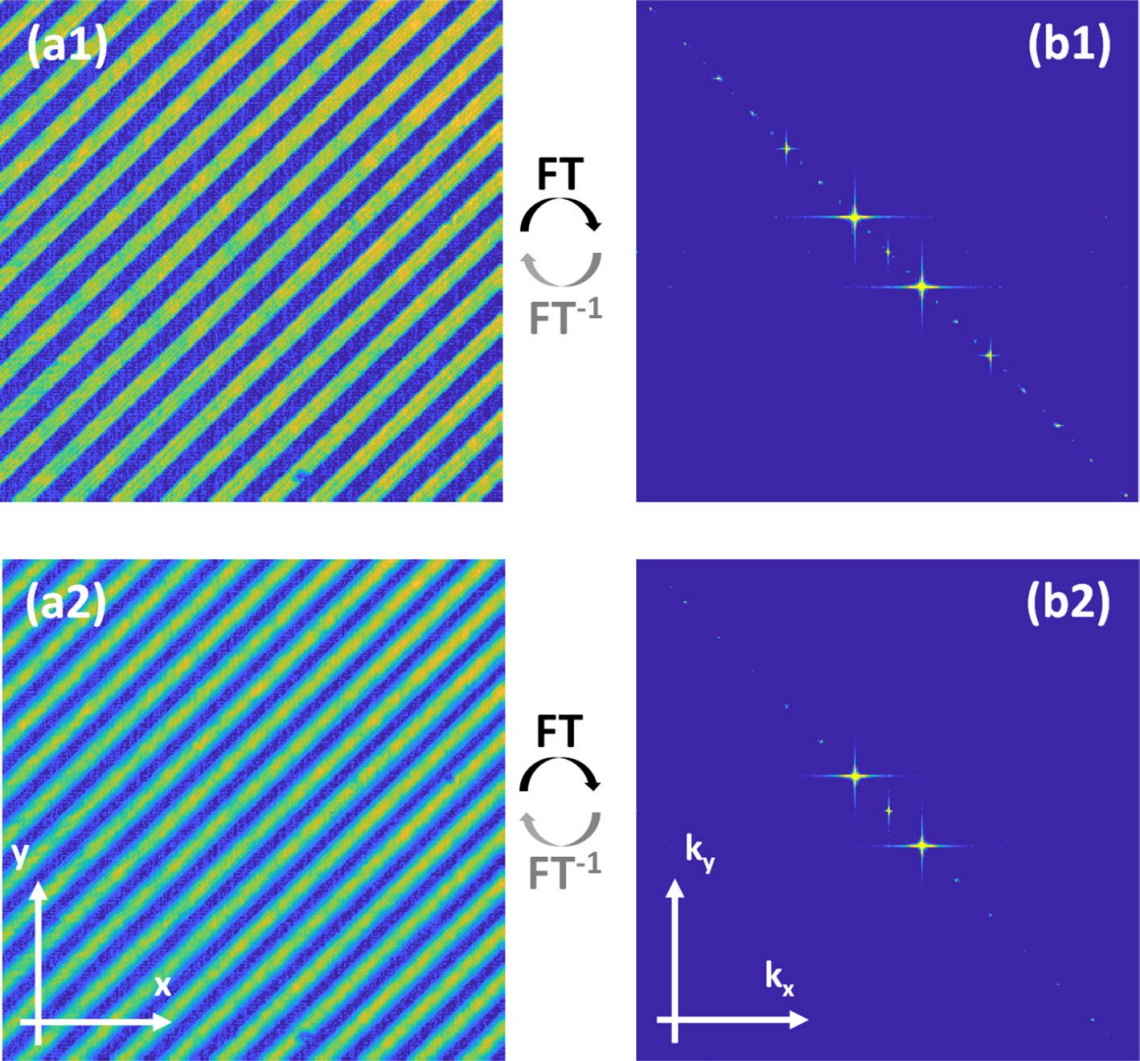
Sinusoidal grating projected from the DMD using amplitude modulation scheme. (**a1**, **a2**) The recorded gratings. (**b1**, **b2**) Fourier transform of recorded gratings. (**a1**, **b1**) show the binary grating and the associated Fourier transform where the diffraction noise (artifacts) are dominant. (**a2**, **b2**) show the sinusoidal grating and the associated Fourier transform in which the 3 main orders are visible with no visible diffraction artifacts.

Figure 9 shows an example of amplitude modulation for a sinusoidal grating with a grating period of 15 pixels displayed onto the DMD and imaged by the camera where it shows the effectiveness of our proposed scheme as compared to binary grating where the higher diffraction orders are visible as shown in Fig. 9(b1). For the amplitude modulation case, higher orders are highly suppressed while the 0th, + 1, − 1 orders are dominant.

**Figure 10:**
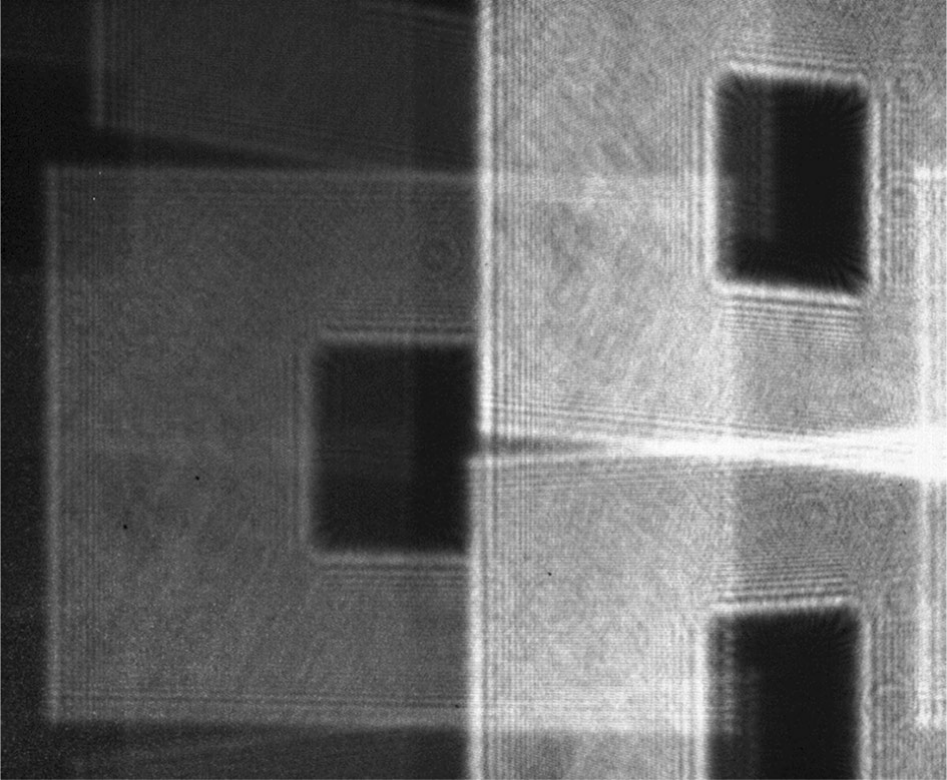
Diffraction patterns from the DMD as a result of mirror deflections.

The DMD consists of individually-controlled mirrors, and this creates many diffraction orders^29^. This phenomenon is shown in Fig. 10 where the pattern recorded on the DMD (a square) is repeated multiple times. The photograph in Fig. 10 was taken by placing the camera close to the DMD in order to capture the multiple patterns and demonstrate the effect.

During the performance of the experiment it was noticed that using a lens (L3 in Fig. 8) with higher focal length showed better reconstructed images. This is due to the fact that as the lens gets far from the DMD, additional diffracted orders from the DMD miss the lens and are not captured by the camera while only the zero-diffracted beam is able to pass through the lens and be captured by the camera. Notice that in practice, this approach is not light efficient, and a lens design such as the one used in display applications^30,31^ that captures all the diffracted orders and superimposes them on the camera is preferable.

**Figure 11:**
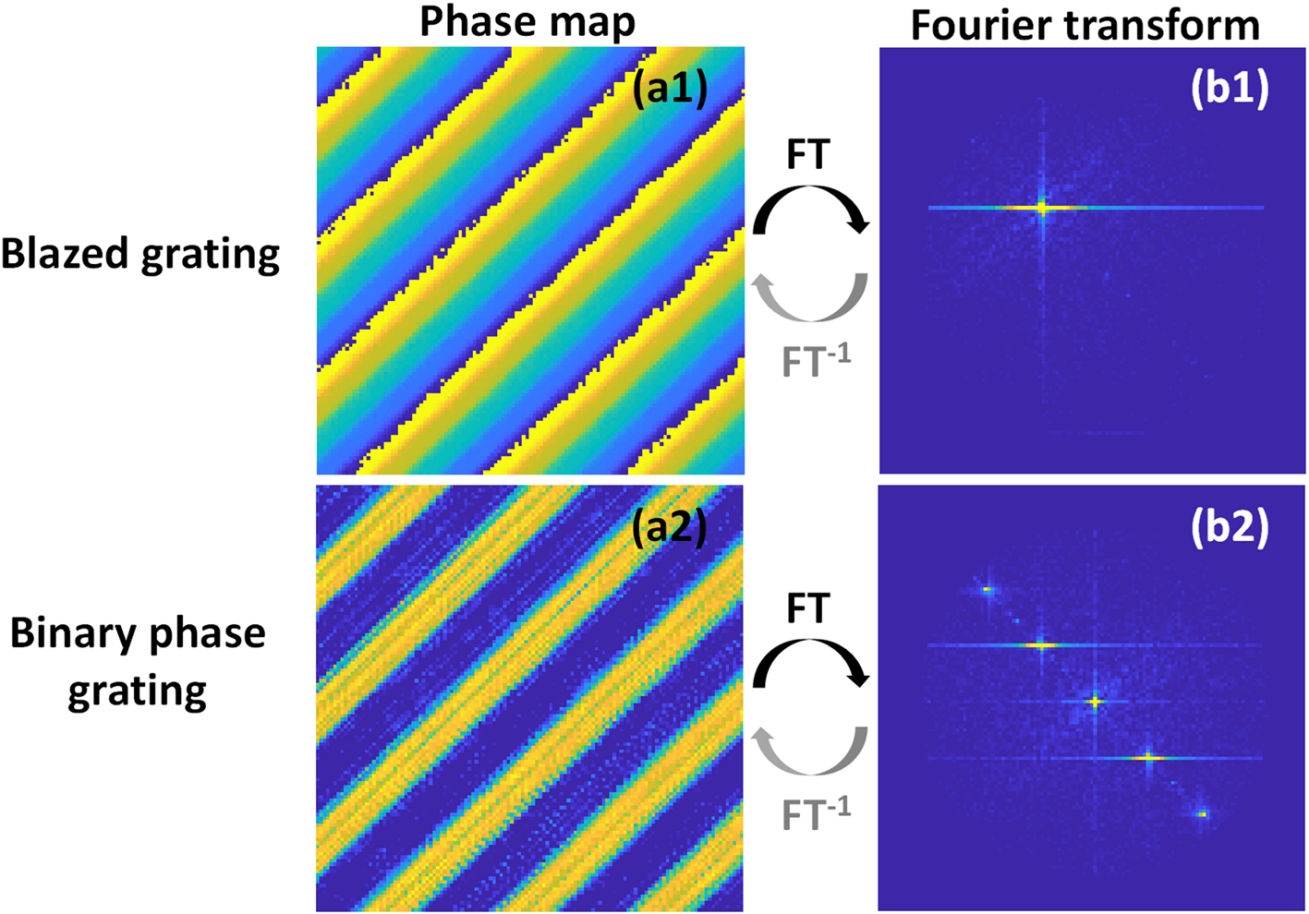
Phase gratings projected from the DMD using amplitude modulation scheme. (**a1**, **a2**) The recorded gratings. (**b1**, **b2**) Fourier transform of recorded gratings. (**a1**, **b1**) show the blazed grating and the associated Fourier transform where the diffraction noise (artifacts) are dominant. (**a2**, **b2**) show the binary phase grating and the associated Fourier transform in which the multiple orders are present.

To further validate our results, phase modulation was performed as described earlier in which the complex field is divided into real and imaginary components and then each component was presented separately using the electro-optic amplitude modulation scheme. We recorded directly intensity on the camera where the signals corresponding to the different bit planes were integrated on the camera. The real and imaginary parts were read-out and were added digitally on the pc to form the complex field. A blazed grating and a binary phase grating were synthesized on the DMD. As shown in Fig. 11, the blazed grating shows all the power focused into the + 1 order. On the other hand, the binary phase grating resulted in multiple orders as expected.

## Discussion

**Figure 12:**
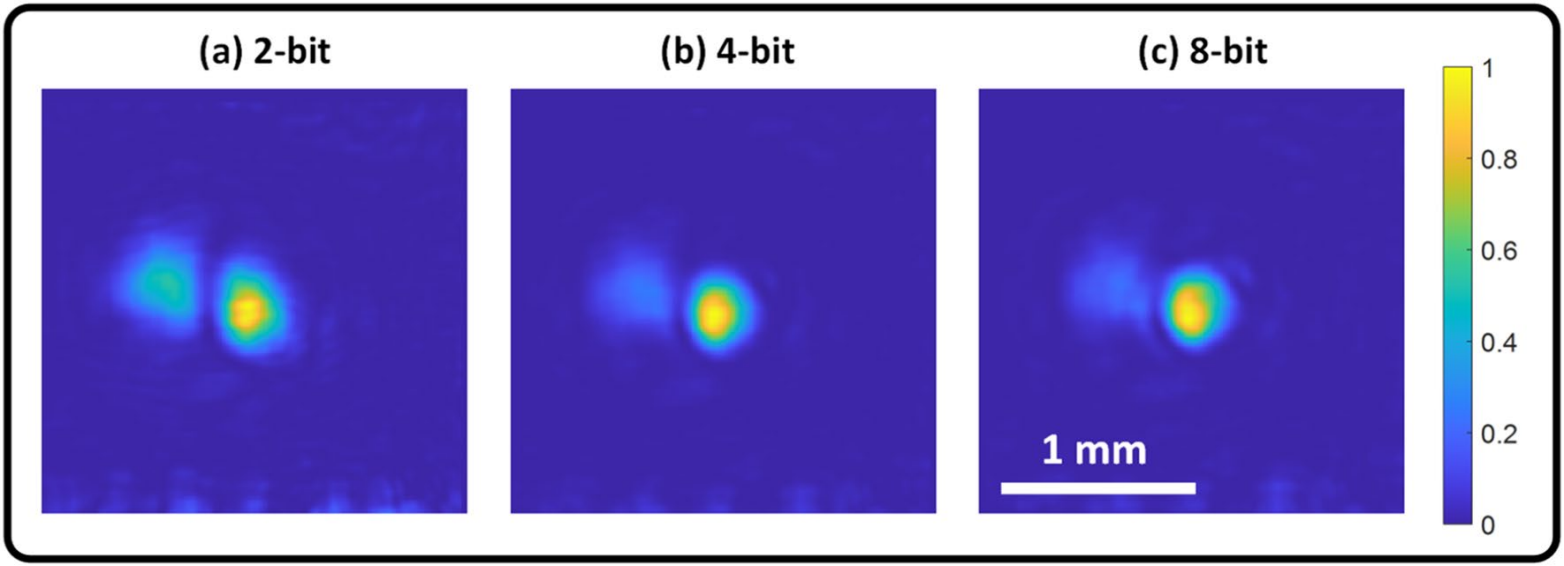
Effect of changing the modulation depth on the focusing quality. (**a**) 2-bit, (**b**) 4-bit, (**c**) 8-bit. (**b**), and (**c**) show well preserved focused spot while (**a**) shows stronger artifacts.

In the optical OEPC experiments, the curvature of the DMD surface was corrected digitally by acquiring another hologram with all the DMD pixels in the “on” state (i.e. background hologram), then all the collected holograms for the real and the imaginary parts were corrected by dividing the extracted fields over the background field, this resulted in a clean image without phase-artifacts as shown in Figs. 2, 3, 4, 5, and 6. For the time-multiplexing scheme, the 8-bit frame rate is rather low (~ 26 frames per second). This speed can be further boosted using a faster commercially available DMD^32^ (refresh rates ~ 32 kHz instead of 6.6 kHz) and also reducing the region of interest (ROI) on the DMD if the application allows a smaller number of pixels. For instance, using the same DMD we used in our study, by decreasing the ROI to 16 × 1140 instead of 912 × 1140, refresh rates can be boosted to 100 kHz (corresponding to a complex-field display rate ~ 110 frames per second). The speed of time modulation technique can be further enhanced by using 4-bit images instead of 8-bit images which will yield a speed enhancement by a factor of 16. Figure 12 shows the effect of changing the number of bits used in the DMD when carrying out OEPC on the focusing quality after passing through the scattering medium (the liver tissue). It is observed that both 4-bit (Fig. 12b) and 8-bit (Fig. 12c) modulation depth resulted in a well preserved focused spot however with a speed enhancement factor of 16 with a display time of 2.4 ms (2.4 ms = 150 µs × 16) with an effective frame rate of 415 frames per second.

Table 1 shows a comparison between amplitude and time modulation schemes in terms of the frame rate for the used DMD whose refresh rate is 6.66 kHz.

**Table 1:** Comparison between different modulation schemes in terms of frame rate/display time.

Modulation\specs	Frame rate	Minimum display time
Binary (1-bit)	$6.6\;{\text{kHz}}$	$150\;\upmu {\text{s}}$
Time (8-bit)	$26\;{\text{Hz}} = \frac{{6.66\;{\text{kHz}}}}{{2^{8} }}$	$38.4\;{\text{ms}} = 150\;\upmu {\text{s}} \times 2^{8}$
Time (4-bit)	$416\;{\text{Hz}} = \frac{{6.66\;{\text{kHz}}}}{{2^{4} }}$	$2.4\;{\text{ms}} = 150\;\upmu {\text{s}} \times 2^{4}$
Amplitude (8-bit)	$833\;{\text{Hz}} = \frac{{6.66\;{\text{kHz}}}}{8}$	$1.2\;{\text{ms}} = 150\;\upmu {\text{s}} \times {8}$
Amplitude (4-bit)	$1665\;{\text{Hz}} = \frac{{6.66\;{\text{kHz}}}}{4}$	$0.6\;{\text{ms}} = 150\;\upmu {\text{s}} \times {4}$

Current DMD technologies have refresh rate as high as 32 kHz which would scale up the modulation speed as shown in Table 2.

**Table 2:** Comparison between different modulation schemes in terms of frame rate/display time for DMD with refresh rate of 32 kHz.

Modulation\specs	Frame rate	Minimum display time
Binary (1-bit)	$32\;{\text{kHz}}$	$31.25\;\upmu {\text{s}}$
Time (8-bit)	$125\;{\text{Hz}} = \frac{{32\;{\text{kHz}}}}{{2^{8} }}$	$8\;{\text{ms}} = 31.25\;\upmu {\text{s}} \times 2^{8}$
Time (4-bit)	$2\;{\text{kHz}} = \frac{{32\;{\text{kHz}}}}{{2^{4} }}$	$0.5\;{\text{ms}} = 31.25\;\upmu {\text{s}} \times 2^{4}$
Amplitude (8-bit)	$4\;{\text{kHz}} = \frac{{32\;{\text{kHz}}}}{8}$	$250\;\upmu {\text{s}} = 31.25\;\upmu {\text{s}} \times {8}$
Amplitude (4-bit)	$8\;{\text{kHz}} = \frac{{32\;{\text{kHz}}}}{4}$	$125\;\upmu {\text{s}} = 31.25\;\upmu {\text{s}} \times {4}$

## Conclusion

High speed, complex wavefront shaping using the digital micro-mirror device was demonstrated. The DMD was used in a time-multiplexing mode which allows for 8-bit grayscale image representation on the DMD within 38.4 ms allowing for focusing in live samples or for other applications without compromising the accuracy. For higher modulation rates, amplitude modulation was demonstrated with maximum achievable frame rate for 8-bit images of 833 Hz yielding a speed up by a factor of 32 as compared to time multiplexing schemes.

The method we demonstrated relies on time integrating holographic recordings of the fields or intensity patterns corresponding to multiple binary patterns on the DMD. The method is particularly useful for optical systems that use digital holography. The method might also be useful in applications where the projected pattern is integrated in time by a light sensitive material such as a fluorescent molecule in imaging or a photo-initiator molecule in 3D printing.

## Supplementary Information

Supplementary Video 1.

## References

[1] Vellekoop IM, Lagendijk A, Mosk AP (2010). Exploiting disorder for perfect focusing. Nat. Photonics.

[2] Yilmaz H, Vos WL, Mosk AP (2013). Optimal control of light propagation through multiple-scattering media in the presence of noise. Biomed. Opt. Express.

[3] Popoff S, Lerosey G, Fink M, Boccara AC, Gigan S (2010). Image transmission through an opaque material. Nat. Commun..

[4] Chaigne T, Katz O, Boccara AC, Fink M, Bossy E, Gigan S (2014). Controlling light in scattering media noninvasively using the photoacoustic transmission matrix. Nat. Photonics.

[5] Yoon J, Lee K, Park J, Park Y (2015). Measuring optical transmission matrices by wavefront shaping. Opt. Express.

[6] Yaqoob Z, Psaltis D, Feld MS, Yang C (2008). Optical phase conjugation for turbidity suppression in biological samples. Nat. Photonics.

[7] Papadopoulos IN, Farahi S, Moser C, Psaltis D (2012). Focusing and scanning light through a multimode optical fiber using digital phase conjugation. Opt. Express.

[8] Hsieh C-L, Pu Y, Grange R, Psaltis D (2010). Digital phase conjugation of second harmonic radiation emitted by nanoparticles in turbid media. Opt. Express.

[9] Ren Y-X, Lu R-D, Gong L (2015). Tailoring light with a digital micromirror device. Ann. Phys..

[10] Akbulut D, Huisman TJ, van Putten EG, Vos WL, Mosk AP (2011). Focusing light through random photonic media by binary amplitude modulation. Opt. Express.

[11] Nam K, Park J-H (2020). Increasing the enhancement factor for DMD-based wavefront shaping. Opt. Lett..

[12] Turpin A, Vishniakou I, Seelig JD (2018). Light scattering control in transmission and reflection with neural networks. Opt. Express.

[13] Zhao T, Ourselin S, Vercauteren T, Xia W (2021). Focusing light through multimode fibres using a digital micromirror device: A comparison study of non-holographic approaches. Opt. Express.

[14] Shin S, Kim K, Yoon J, Park YK (2015). Active illumination using a digital micromirror device for quantitative phase imaging. Opt. Lett..

[15] Bianchi S, Di Leonardo R (2012). A multi-mode fiber probe for holographic micromanipulation and microscopy. Lab Chip.

[16] Turtaev S, Leite IT, Mitchell KJ, Padgett MJ, Phillips DB, Čižmár T (2017). Comparison of nematic liquid-crystal and DMD based spatial light modulation in complex photonics. Opt. Express.

[17] Conkey DB, Caravaca-Aguirre AM, Piestun R (2012). High-speed scattering medium characterization with application to focusing light through turbid media. Opt. Express.

[18] Lee W-H (1979). Binary computer-generated holograms. Appl. Opt..

[19] Goorden SA, Bertolotti J, Mosk AP (2014). Superpixel-based spatial amplitude and phase modulation using a digital micromirror device. Opt. Express.

[20] Drémeau A, Liutkus A, Martina D, Katz O, Schülke C, Krzakala F, Gigan S, Daudet L (2015). Reference-less measurement of the transmission matrix of a highly scattering material using a DMD and phase retrieval techniques. Opt. Express.

[21] Drémeau, A. & Krzakala, F. Phase recovery from a Bayesian point of view: The variational approach. In Proceedings of IEEE Transactions on Acoustics, Speech, and Signal Processing (2015).

[22] Wang D (2015). Focusing through dynamic tissue with millisecond digital optical phase conjugation. Optica.

[23] Ren Y-X, Li M, Huang K, Wu J-G, Gao H-F, Wang Z-Q, Li Y-M (2010). Experimental generation of Laguerre–Gaussian beam using digital micromirror device. Appl. Opt..

[24] Gong L, Ren Y-X, Xue G-S, Wang Q-C, Zhou J-H, Zhong M-C, Wang Z-Q, Li Y-M (2013). Generation of nondiffracting Bessel beam using digital micromirror device. Appl. Opt..

[25] Adeyemi AA, Barakat N, Darcie TE (2009). Applications of digital micro-mirror devices to digital optical microscope dynamic range enhancement. Opt. Express.

[26] Ding X, Ren Y, Lu R (2015). Shaping super-Gaussian beam through digital micro-mirror device. Sci. China Phys. Mech. Astron..

[27] Jin D, Zhou R, Yaqoob Z, So PTC (2017). Tomographic phase microscopy: Principles and applications in bioimaging. J. Opt. Soc. Am. B.

[28] Lee K, Kim K, Kim G, Shin S, Park YK (2017). Time-multiplexed structured illumination using a DMD for optical diffraction tomography. Opt. Lett..

[29] Park M-C, Lee B-R, Son J-Y, Chernyshov O (2015). Properties of DMDs for holographic displays. J. Mod. Opt..

[30] Pan J-W, Wang H-H (2013). High contrast ratio prism design in a mini projector. Appl. Opt..

[31] Pan J-W, Wang C-M, Sun W-S, Chang J-Y (2007). Portable digital micromirror device projector using a prism. Appl. Opt..

[32] Texas Instruments Incorporated. DLP7000. https://www.ti.com/product/DLP7000.

